# Diagnostic and Therapeutic Challenges of a Large Tongue Lymphangioma in a Child: A Case Report

**DOI:** 10.7759/cureus.47363

**Published:** 2023-10-20

**Authors:** Dimitrios Andreadis, Alida Ndreu, Antonios Tsekos, Prashanth Panta

**Affiliations:** 1 Oral Medicine/Pathology, Aristotle University of Thessaloniki, Thessaloniki, GRC; 2 Oral and Maxillofacial Surgery, Agios Loukas Hospital, Thessaloniki, GRC; 3 Oral Medicine and Radiology, Malla Reddy Institute of Dental Sciences, Hyderabad, IND

**Keywords:** lymphatic system, embolization, lymphangiomas, malformation, tongue

## Abstract

Lymphangiomas are rare, painless, benign tumors in infancy or early childhood resulting from a congenital malformation of the lymphatic vessels with variable clinical appearance. We report the case of a six-year-old male child who presented with a micronodular surface of the tongue, a burning sensation, difficulties during swallowing and mastication, as well as speech disturbances. Histological examination of the excised tongue specimen showed an angiomatous lesion of the lamina propria that comprised many wide thin-walled spaces. MRI examination revealed an area of disparate T2 signal maximum diameter of 2-3 cm with cystic texture in the middle and frontal left part of the tongue. The lesion was diagnosed as a lymphangioma and was managed through pre-operative embolization and surgery. Tongue lymphangiomas may lead to aesthetic problems, functional issues (like dysphagia, airway obstruction, and speech difficulties), psychological disturbances, poor oral hygiene, and occasional bleeding associated with oral trauma. A major fraction of patients also experience infections, often leading to a significant increase in lesion size. A combination of preoperative embolization and surgical excision could be chosen considering its large size and the age of the patient, and to further eliminate the possibility of recurrence. Early diagnosis and radical treatment are critical in its management.

## Introduction

Lymphangiomas consist of rare developmental lesions of the lymphatic vessels, mainly found in children under five years of age. They are present as micro-cysts or lobulated masses and are commonly located in the head or neck area. They were described by Redenbacher in 1828 for the first time and in 1854, Virchow described the first tongue lymphangiomas [1]. Lymphangiomas are characterized as malformations rather than true neoplasms. The etiology includes viral infection, use of addictive substances or drug administration during pregnancy, and toxic environmental factors. Lymphangiomas may also constitute part of syndromes including Turner, Noonan's syndromes, etc. [2]. It has been considered to develop from primitive embryonic lymphatic origin. These lymphatic areas never develop anastomoses with the larger lymphatic vessels and therefore, it leads to the formation of localized lymphatic structures so-called lymphangiomas or cystic hygromas [3].

Sabin et al., in 1902, proposed that the lymphatic system is derived from primordial endothelial buds originating from the developing venous system starting at 6.5 weeks of gestation. These buds coalesce to form plexi at the neck. Peripheral malformations arise from abnormal plexus networks in the periphery of the body [4]. There are three theories; the first theory suggests the blockage of normal growth of the primitive lymph channel during embryogenesis, the second theory suggests that the primitive lymph sac does not reach the venous system, and the third theory suggests the ectopic presence of lymph tissues during embryogenesis [5].

Generally, there is no sex predilection, but cases of small-sized lymphangiomas (< 1 cm) of the alveolar mandible ridge show a 2:1 male-to-female ratio [6]. The frequency of lymphangiomas ranges from 1.2 to 2.8 per 1000 newborns [3]. Almost 50% of the cases are observed at birth, and 90% develop by the age of two. The diagnosis can range from the fifth month of gestation to the second decade of life. Lymphangiomas are mostly seen in the head and neck region, which accounts for about 75% of all cases. In the oral cavity, gingiva, buccal mucosa, lips, and the anterior dorsal surface of the tongue are the most common sites. Oral lymphangiomas constitute 6% of all lymphangiomas [7,8]. Clinically, tongue lymphangiomas consist of multiple blister-like nodules of the anterior two-thirds of the dorsal surface of the tongue, which becomes enlarged. In cases of large lymphangiomas, these lesions may cause macroglossia. 

According to De Serres et al., lymphatic malformations can be classified into five groups ranging from single superficial microcystic lymphatic malformation of the tongue (stage I) to extensive microcystic lymphatic malformations involving larger areas of the tongue, floor of the mouth, and further cervical structures (stage V) (Stage 1: Unilateral infrahyoid, Stage 2: Unilateral suprahyoid, Stage 3: Unilateral infrahyoid and suprahyoid, Stage 4: Unilateral suprahyoid, and Stage 5: Bilateral infrahyoid and suprahyoid). The large extension of lymphangiomas may lead to aesthetic, functional, occlusal, psychological disturbances, poor oral hygiene, and bleeding from the tongue associated with oral trauma. About 70-80% of patients with lymphatic malformation experience infections, which are often associated with a significant increase in the size of the lesions [9]. Early diagnosis and treatment help reduce the functional, psychological, and cosmetic damage [10].

## Case presentation

A six-year-old male patient presented with a micronodular, enlarged, surface of the tongue, burning sensation, difficulties during mastication and swallowing, difficulties in speech, irregular bleeding of the tongue, and quite poor oral hygiene. According to his parents, the tongue was apparently normal until the age of three years, after which a swelling, that was slowly growing, was observed. In the beginning, it appeared as a painless soft mass that gradually enlarged and then remained stable for a long period. Neither facial asymmetry nor other anatomic head and neck malformation was observed. All these symptoms had a big impact on the patient’s quality of life. The size of the tongue was normal, about 8 cm. There was no relevant family history. The clinical examination revealed a hyperplastic overgrowth extending to the anterior two-thirds of the dorsal surface of the tongue characterized by small soft irregular vesicles with a red-brownish-yellow color. The child was in good general condition, without signs of obstruction of the superior respiratory tract.

The differential diagnosis included hemangioma, lymphoepithelial cyst, mesenchymal neoplasms, vascular malformations, and lipoma. A visceral skull MRI was performed with a 1.5T MRI (Siemens Avanto; Siemens Healthineers, Erlangen, Germany). Incisions were made with the burden of T1 and T2 time before and after the intravenous administration of the paramagnetic contrast medium. The MRI examination revealed an area of disparate T2 signal maximum diameter of 2.3 cm with cystic texture in the middle and frontal left part of the tongue. These findings could be likely related to lymphangioma and less likely to hemangioma. The major salivary glands and the base of the oral cavity appeared to be normal. No pathological signs such as enlargement of lymph nodes of the neck were observed.

**Figure 1:**
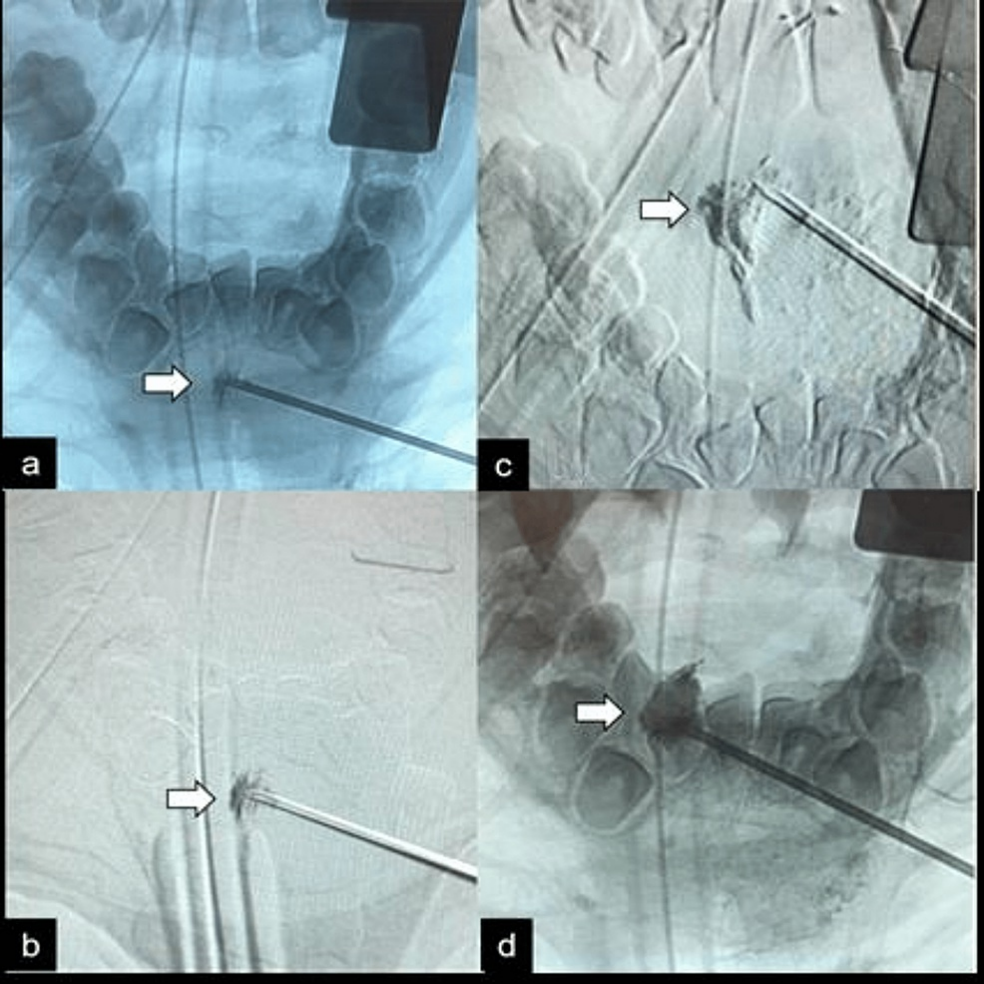
Preoperative direct embolization of lymphangioma with sclerosing agent. The fluoroscopic images (a to d) demonstrate a gradual filling of the lymphangioma with a sclerosing agent (arrows).

**Figure 2:**
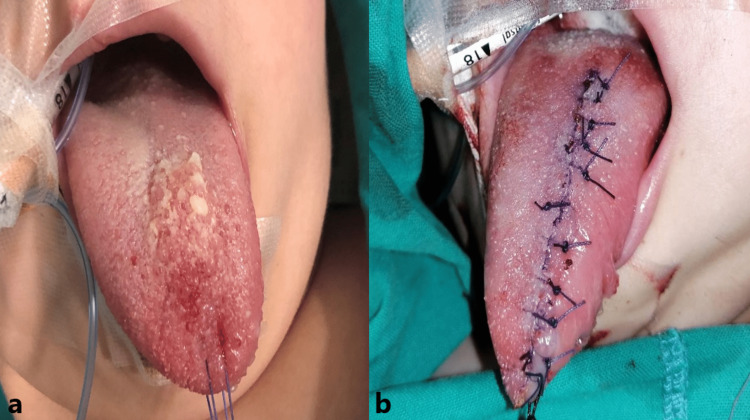
Clinical pictures before and after the surgical excision of the lymphangioma

**Figure 3:**
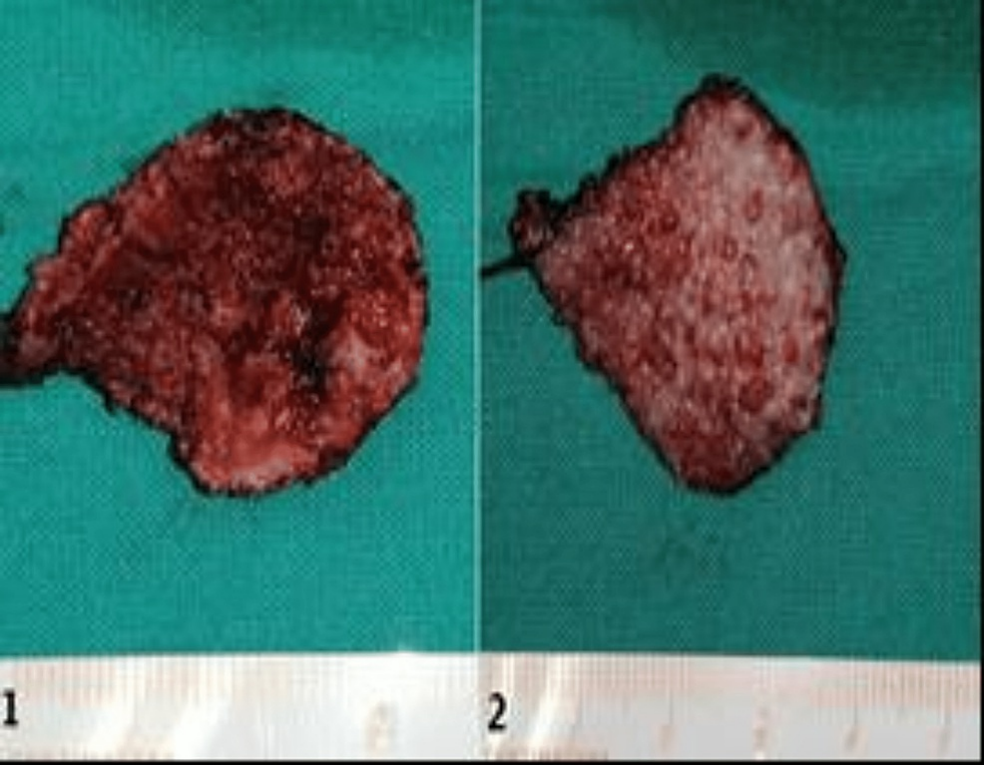
The extracted lesion of the tongue

The treatment options were surgical removal, laser ablation with long-pulsed neodymium-doped yttrium aluminum garnet (Nd-YAG) or carbon-dioxide (CO2) laser, embolization, radiofrequency or sclerotherapy, interferon, and corticosteroids. Cryotherapy and laser can be considered for superficial and localized lymphangiomas. Due to the lesion's large size and the location of the damage, surgical excision with preoperative embolization was chosen as a treatment option. The decision was made after carrying out the necessary blood tests and the MRI. No immediate interventions were under consideration. Analytically, the immediate preoperative direct embolization with the sclerosing agent, Sodium tetradecyl sulfate (the most common sclerosing agent), was performed (Figure 1). Afterward, the abnormal surface of the tongue was surgically removed under general anesthesia (Figure 2). The size of the removed tongue was 4 cm (Figure 3). The postoperative course was good without any complications.

**Figure 4:**
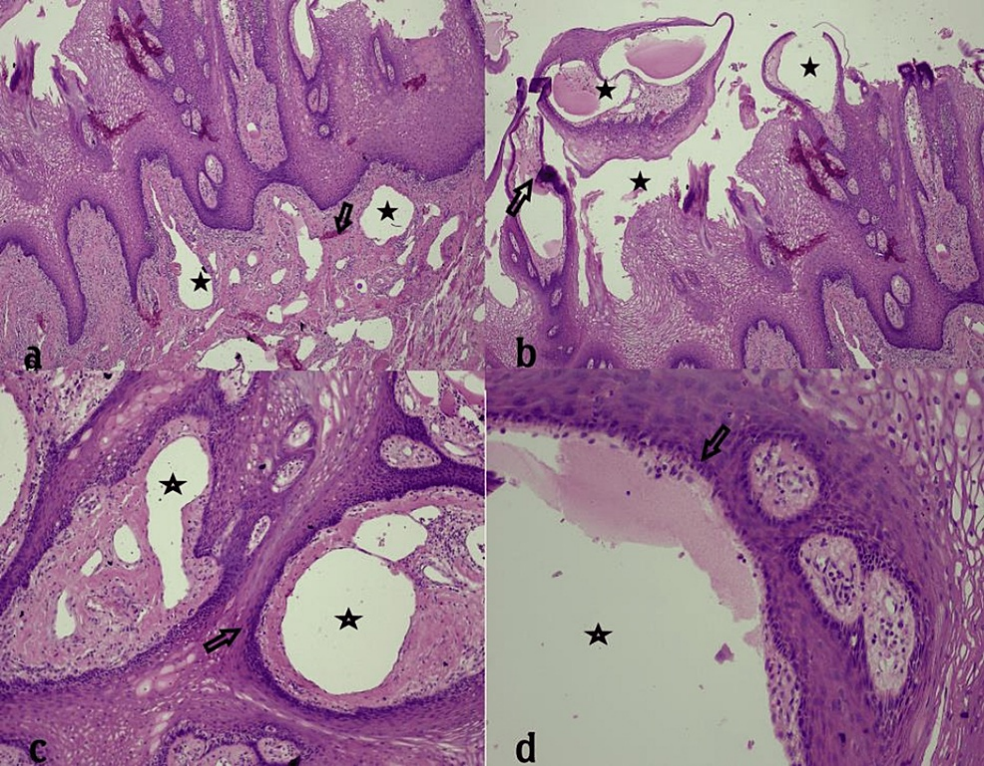
Histological findings. Magnification power in a, b, and c is 10x, and 100x in d.

Histological examination (on hematoxylin & eosin-stained sections) of the tongue excision specimen showed multiple vascular lesions of the lamina propria that comprised many wide thin-walled spaces, lined by a row of endothelial cells without atypia. Some of them were filled with red blood cells. On immunohistochemical stain for antigen D2-40, the endothelial cells were positive. In epithelium, the vascular spaces aggregated in clusters and were separated by delicate fibrous bands (septa). A few vascular spaces extended to the subjacent striated muscle fibers. Mild inflammatory cells infiltrating the stroma, consisting of lymphocytes and plasma cells, were also found. The combined clinical, imaging, and microscopic findings settled the final diagnosis of an oral (tongue) lymphangioma (Figure 4) (De Serres-stage I). 

During the six-month follow-up, no recurrence was observed (Figure 6). The subsequent follow-up plan was to see the patient every year after the operation until a teenager.

**Figure 5:**
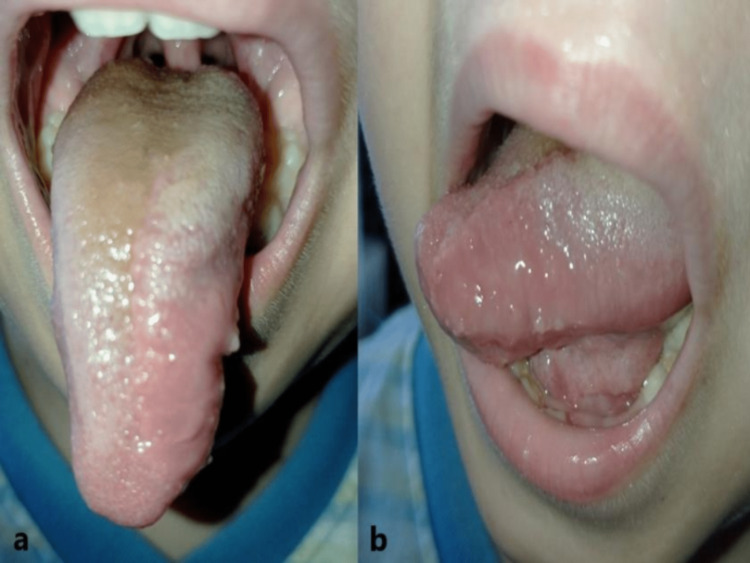
Postoperative recovery of the tongue

## Discussion

The rarity of lymphangiomas and their preference for the head/neck area and oral cavity is characteristic [4-5,10-13]. The origin of this entity is unknown. It has been hypothesized that the blockage of normal growth of the primitive lymph channels during embryogenesis, a primitive lymphatic structure that cannot be linked to the main lymphatic system, or a possible ectopic lymphatic tissue development during embryogenesis can explain their origin [9,14]. Lymphangiomas are classified as microcystic (capillary lymphangiomas), macrocystic acrocystic lymphangiomas, and cystic hygromas according to the size of the lymphatic cavities [14]. When a lymphangioma is located at the tongue site, as in our case, it presents as a so-called cavernous lymphangioma. When it develops in the neck, it usually occurs as a cystic lesion, so-called cystic hygromas (approximately 90% of the lymphangiomas are in the head and neck area).

Lymphangioma of the tongue reveals a granular appearance on the surface, microcysts with a thin wall containing lymph, microscopically. Their color ranges from translucent to red. Tongue lesions are mostly slow-growing but some develop quickly, as in our case [2]. Occasionally, lymphangiomas contain lymphatic vessels with a marked dilation (cavernous lymphangioma) or macroscopic cyst-like structures (cystic hygroma) [6-8]. The vessels often infiltrate the adjacent soft tissues, and they contain proteinaceous fluid, intermixed with sparse lymphocytes and red blood cells. Whether the presence of red blood cells represents a secondary hemorrhage into a lymphatic vessel or mixed lymphangioma and hemangioma is not clear. In intraoral tumors, the lymphatic vessels are located immediately under the oral epithelium. The superficial location results in a translucent, vesicle-like clinical appearance, but lymphangiomas can be seen deeper in the connective tissue and muscles.

Despite the large size of the lesion in the current case, the tongue was only superficially involved. Overall, the capillary lymphangioma consists of small vessels, in contrast to the cavernous lymphangioma with larger lymph channels and the cystic lymphangioma with large macroscopic cystic spaces. The lymphatic endothelium reveals positive staining for marker D2-40. Small blood vessels positively stained for CD31 are also present in the lamina propria [9,14,15]. Prenatal chromosomal analysis including 13, 18, 21, X, and Y may reveal the increased possibility for lymphangioma formation. In addition, growth factors such as vascular endothelial growth factor C (VEGF-C) and its receptors may play a critical role in lymphatic malformations in this period [8,9,14]. Lymphangiomas are part of several syndromes, including Turner's syndrome, Noonan's syndrome, and Maffucci's syndrome [2,3,6,16].

The clinicopathologic differential diagnosis for lymphangioma includes vascular malformations, hemangioma, amyloidosis, lipoma, congenital hypothyroidism, neurofibromatosis, papillary hyperplasia of the palate, lymphoepithelial cyst, lingual thyroid, pyogenic granuloma, other mesenchymal neoplasms, and oral papillomatosis. In contrast to hemangiomas, spontaneous regression of lymphangiomas is rare, but it usually presents as a slow progressive enlargement. Hemangiomas are vascular tumors that can appear similar to lymphangiomas, but they are composed of blood vessels rather than lymphatic vessels. Vascular malformations are abnormal collections of veins that can sometimes be confused with lymphangiomas especially when they include deeper tissues. Cystic hygromas are fluid-filled sacs that typically occur in the head and neck region. Lipomas are fatty tumors sometimes similar to soft tissue lymphangiomas, characterized by painless and slow growth. Both benign and malignant tumors can manifest similarly to lymphangiomas [3,16]. The treatment of lymphangiomas depends on their type, size, site, and infiltration of the surrounding tissues. Microcystic lymphangiomas can be diffuse and thus difficult to eradicate, while macro cystic lesions are localized and easily excised. 

Total removal may not be possible in all cases, depending on the size or involvement of vital structures. No treatment for non-enlarging lymphangiomas of the tongue has been also proposed due to the high recurrence rate. USG, CT, and MRI can be used to define the location of the lesion in relation to the neighboring structures involving the muscle [9]. Surgical excision, radiation therapy, cryotherapy, electrocautery, sclerotherapy, steroid administration, embolization, ligation, Nd-YAG/CO2 laser surgeries, and radiofrequency tissue ablation have been performed; however, surgical excision of localized lymphangiomas remain the best option [16,17]. Embolization can be very helpful for managing lymphangiomas as in the current case because they cause a localized inflammatory reaction, obliterative thrombosis of hemangiomas/lymphangiomas, subsequent fibrosis of the endothelial spaces, and regression of the lesion [17].

## Conclusions

Tongue lymphangiomas may lead to aesthetic problems, functional issues, psychological disturbances, poor oral hygiene, and occasional bleeding. A combination of preoperative embolization and surgical excision could be chosen in younger patients with large lesions. In the present report, preoperative embolization and surgical excision gave ideal results. Although lymphangiomas are relatively common, there is a need for more literature about the efficacy of embolization in young patients. Long-term follow-up is necessary for early detection of possible relapse. Early diagnosis and radical treatment are effective. Also due to variability in clinical presentation and the potential overlap of symptoms with other conditions, it is crucial for the healthcare provider to perform a thorough evaluation which may include imaging studies MRI, CT scans, and biopsy to accurate diagnoses and determine the appropriate treatment plan for patients. Consulting with specialists such as oral maxillofacial surgeons and radiologists can be beneficial in complicated cases.
